# Investigation of the molecular network underlying PET-MPs-induced inflammatory bowel disease via integrated machine learning and molecular docking approaches

**DOI:** 10.1038/s41598-026-57094-0

**Published:** 2026-06-14

**Authors:** Jinhua Ye, QianYu Lu, Hufei Wang, Zhiyong Chen, Songlin Ran

**Affiliations:** 1 Department of Gastroenterology, Suizhou Hospital, Hubei University of Medicine, Suizhou, China; 2 Department of Nephrology, Zhongnan Hospital of Wuhan University, Wuhan, China; 3 Department of Gastrointestinal Surgery, The First Affiliated Hospital, Sun Yat-sen University, Guangzhou, Guangdong China

**Keywords:** Inflammatory bowel disease, Machine learning, Molecular docking, Microplastics, Network toxicology, Computational biology and bioinformatics, Diseases, Gastroenterology

## Abstract

Polyethylene terephthalate microplastics (PET-MPs), as environmental contaminants, have raised significant concerns due to their potential toxicity, leading to serious environmental pollution and health issues. However, their impact on inflammatory bowel disease (IBD) remains poorly elucidated. This study aims to investigate the potential molecular mechanisms linking PET-MPs to IBD pathogenesis. Multiple datasets were employed to identify Crohn’s disease (CD)- and ulcerative colitis (UC)-associated targets. Multi-machine learning approaches were combined with molecular docking of a PET-related compound to evaluate potential binding interactions with the identified hub targets. This study delineated 9 putative targets associated with PET-MPs-related CD pathogenesis and 17 potential targets associated with UC pathogenesis. For CD, GBM showed the best performance among all models, with a mean ROC-AUC of 0.862; for UC, RF performed best, with a mean ROC-AUC of 0.806, based on both training and validation sets. Multi-machine learning analysis identified 14 hub genes as candidate key regulatory factors. SHAP analysis highlighted their significant contributions to the model predictions. Molecular docking simulations suggested favorable binding affinities between a PET-related compound and the hub targets. This study suggests that PET-MPs may contribute to IBD pathogenesis by potentially interacting with 14 machine learning-prioritized hub genes. Molecular docking analyses indicated predicted high-affinity binding between a PET-related compound and these targets. Collectively, these findings provide a hypothesis-generating framework for investigating the potential role of PET-MPs in IBD progression.

**Supplementary Information:**

The online version contains supplementary material available at https://doi.org/10.1038/s41598-026-57094-0.

## Introduction

Inflammatory bowel disease (IBD) refers to a group of recurrent, chronic inflammatory disorders of the gastrointestinal tract with unknown etiology and pathophysiology, encompassing ulcerative colitis (UC) and Crohn’s disease (CD)^1^. IBD ranks among the most prevalent gastrointestinal diseases globally, and its incidence continues to rise worldwide^2^. Consequently, the associated economic and public health burdens are increasing. However, the precise etiology and pathogenesis of IBD remain incompletely understood, posing significant obstacles to its prevention and treatment. Current perspectives indicate that IBD pathogenesis involves genetic, environmental, and immune factors, among which environmental contributors are receiving growing attention.

Microplastics (MPs), recognized as persistent, bioaccumulative, and toxic emerging global pollutants, are increasingly evidenced to exert adverse effects on human health. Humans are exposed to MPs via inhalation, ingestion, and dermal contact, with ingestion through the digestive tract serving as the primary exposure route^3^. MPs are now widely detected in human feces, organ tissues, and blood. Converging evidence from ecological, epidemiological, and mechanistic studies suggests a significant association between MPs exposure and both intestinal inflammation and IBD^4–6^. MPs have been shown to compromise intestinal barrier function, potentially facilitating their penetration through the gut mucus layer and even the epithelial cell layer. This may trigger a cascade of adverse consequences, including oxidative stress, inflammatory responses, impaired immune function, and tissue degeneration—all recognized factors associated with IBD pathogenesis. Polyethylene terephthalate (PET), valued for its superior mechanical strength, chemical stability, and transparency, is extensively used in food and beverage packaging, textile fibers, and medical devices. Notably, one study reported significantly higher concentrations of PET microplastics (PET-MPs) in the feces of IBD patients compared to healthy controls^7^.

Despite these advances, current research remains insufficient, and the underlying mechanisms linking MPs exposure to IBD are still unclear. Crucially, the potential impact of PET-MPs on the multi-scale molecular networks underlying IBD has been largely unexplored. To address this gap, our study employs multi-omics data to construct a potential toxicological network linking PET-MPs to IBD pathogenesis. By integrating network toxicology and functional enrichment analysis with multiple machine learning algorithms^8^, we built models to identify and prioritize candidate hub targets. Subsequent molecular docking of a PET-related compound was performed to evaluate potential binding interactions and binding energies with the identified hub targets. Through an integrated application of these strategies, this study aims to explore the potential mechanistic role of PET-MPs in IBD pathogenesis. Our work provides a hypothesis-generating framework for future investigation of PET-MPs toxicity and molecular mechanisms, may offer supportive insights for the development of IBD chemoprevention and precision therapeutic strategies, and contributes to addressing the unmet clinical needs of IBD patients.

## Methods

### Acquisition of disease-associated targets

Eight inflammatory bowel disease (IBD) transcriptomic datasets (GSE20881^9^, GSE48634^10^, GSE75214^11^, GSE102133^12^, GSE179285^13^, GSE11223^14^, GSE206285^15–17^, and GSE92415^18^) were retrieved from the NCBI GEO database. In the analysis of Crohn’s disease (CD), datasets GSE20881 and GSE179285 were merged to form the discovery cohort, while the other three datasets (GSE48634, GSE75214, and GSE102133) were designated as the validation cohort. In the analysis of ulcerative colitis (UC), datasets GSE11223 and GSE206285 were merged to form the discovery cohort, while the other three datasets (GSE48634, GSE92415, and GSE179285) were designated as the validation cohort (Table S1).

**Fig. 1:**
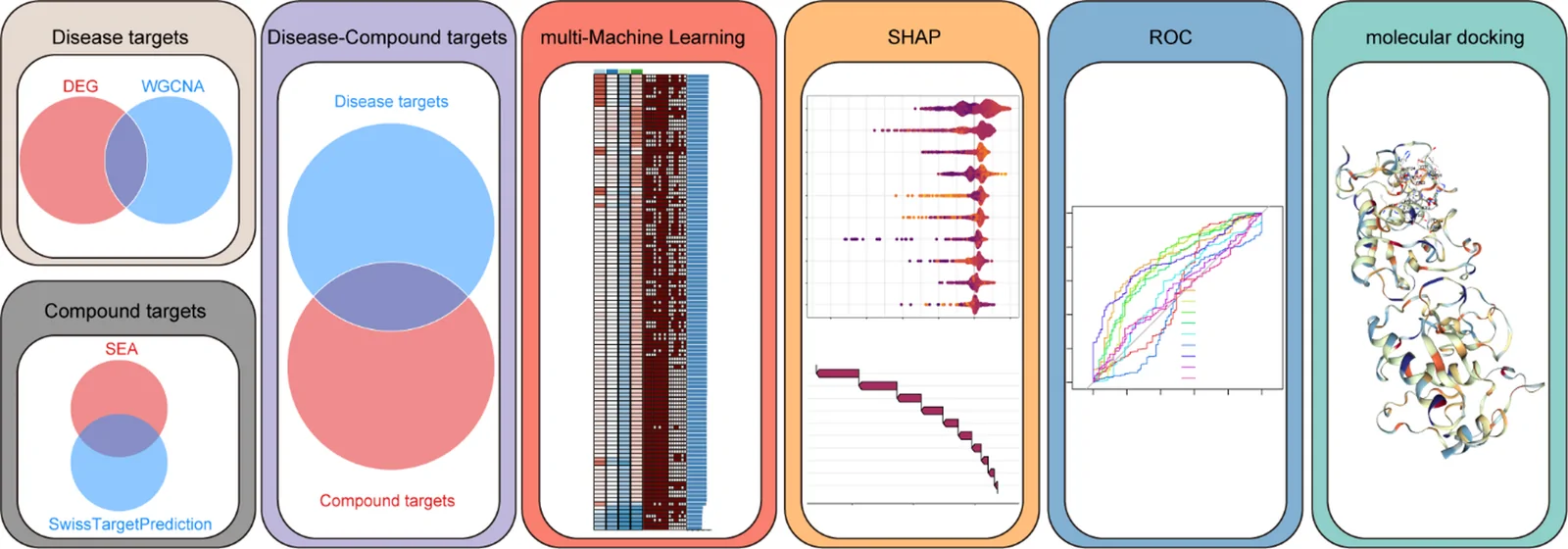
Flowchart of this article.

Batch effects were mitigated using the sva packages. To evaluate the effectiveness of batch effect removal, Principal Component Analysis (PCA) was employed to compare clustering patterns of inter-batch samples before and after correction. The complete analytical workflow is depicted in Fig. 1.

### Elucidation of PET chemical structure and biological targets

The canonical two-dimensional structural descriptor (SMILES) of bis(2-hydroxyethyl) terephthalate (PET) was obtained from the PubChem database^19^. Potential biological targets of PET were predicted using the SwissTargetPrediction^20^ and Similarity Ensemble Approach (SEA)^21^ databases. Protein-protein interaction (PPI) networks for the targets were constructed using the STRING database^22^ and visualized with Cytoscape software^23^(v3.10.3).

### Differential gene expression analysis

We performed analysis of GEO transcriptomic data using the limma package. Differentially expressed genes (DEGs) for Crohn’s disease (CD) were identified using thresholds of adjusted *P* < 0.05 and |log2FC| > 0.263 (corresponding to a 1.2-fold change). DEGs for ulcerative colitis (UC) were identified using thresholds of adjusted *P* < 0.05 and |log2FC| > 0.585 (corresponding to a 1.5-fold change). Results were visualized using ggplot2.

### Weighted gene co-expression network analysis (WGCNA)

Gene co-expression networks were constructed using the WGCNA package^24^. The normalized expression matrix was filtered to retain genes with standard deviation > 0.5 across samples. Sample outliers were removed by hierarchical clustering (Euclidean distance, average linkage) with a height cutoff of 20,000. Clinical trait data (Control/Treat) were integrated by matching samples between expression and phenotype datasets. A soft-thresholding power (β = 6) was selected where the scale-free topology fit reached R^2^ ≥ 0.8. The adjacency matrix was transformed into a Topological Overlap Matrix (TOM) to minimize spurious connections, and modules were identified via hierarchical clustering with a minimum size of 60 genes. Modules with eigengene correlation > 0.75 were merged. Module-trait associations were assessed by correlating module eigengenes with clinical traits (Pearson correlation). Intramodular hub genes were identified using gene significance (|cor(gene, trait)|) and module membership (|cor(gene, module eigengene)|), with thresholds of MM > 0.8 and GS > 0.2. Key modules were visualized in trait relationship heatmaps and scatterplots of GS versus MM relationships.

### Identification of PET-associated disease targets

Intersection analysis between disease-associated targets (derived from the union of differentially expressed genes, DEGs, and WGCNA hub genes) and computationally predicted PET targets identified PET-relevant disease targets. The overlapping targets were visualized using Venn plots.

### Functional enrichment analysis

Gene Ontology (GO) analysis (covering biological processes, cellular components, and molecular functions) and Kyoto Encyclopedia of Genes and Genomes (KEGG)^25^ pathway enrichment were performed using the DAVID database^26^(*P* < 0.05). Results were visualized through the SangerBox online platform^27^.

### Machine learning-based screening of hub genes

We employed an integrated machine learning framework incorporating multiple algorithms to systematically identify hub disease targets associated with PET. Specifically, GEO expression profiles served as the training dataset, enabling comparative evaluation of 113 distinct predictive models generated through diverse combinations of 12 classical machine learning algorithms (Lasso, Stepglm, SVM, glmBoost, Ridge, Enet, plsRglm, RF, GBM, LDA, XGBoost, and NaiveBayes)^28^. Hub targets were subsequently selected based on optimal model performance. Model performance was rigorously evaluated based on the area under the receiver operating characteristic curve (AUC). Models were subsequently ranked according to their mean AUC values. The top-performing model was selected, yielding candidate hub genes identified by this optimal model.

### Model interpretation

We implemented the SHAP (SHapley Additive exPlanations) algorithm to quantitatively assess feature importance. This method computes the SHAP value for each feature, thereby enabling interpretable quantification of its contribution to model predictions. We leveraged the shapviz package to compute SHAP values and visualize the results.

### Molecular docking

Molecular docking simulations were employed to computationally validate interactions between PET and hub genes associated with Crohn’s disease (CD) and ulcerative colitis (UC). The ligand structure (PET) in SDF format was retrieved from PubChem^19^. Receptor structures of core targets in PDB format were obtained from the AlphaFold Database and Protein Data Bank (PDB)^29^. Molecular docking of PET with core targets was performed using the CB-Dock2 online platform^30,31^, with the number of cavities for docking parameter set to 10. The optimal conformation exhibiting the highest binding affinity was visualized for analysis.

## Results

### Identification of PET targets

**Fig. 2:**
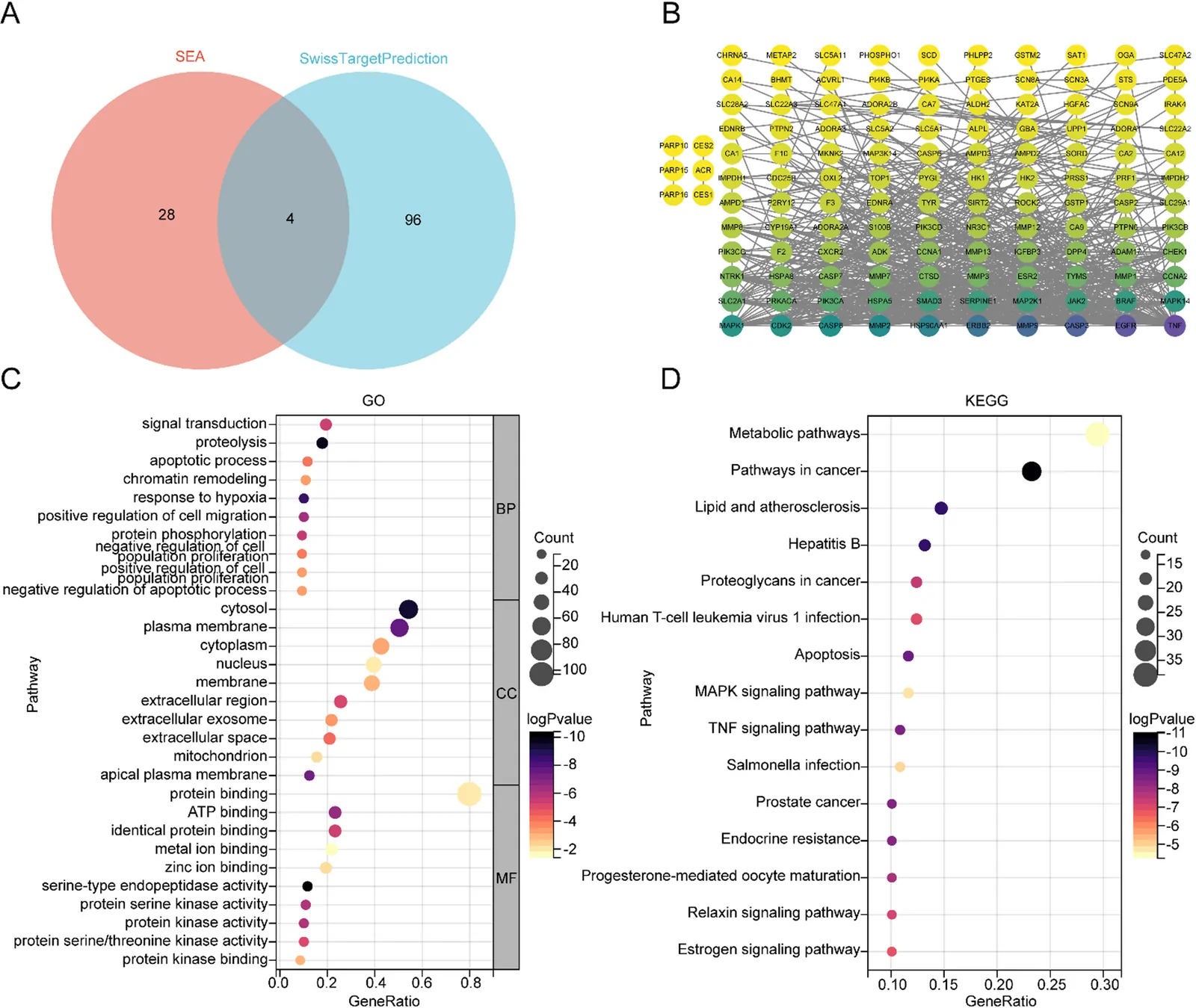
Identification of the targets for PET. (**A**) Venn Diagram Visualization of Predicted Target. (**B**) Visualization of PET target interactions using a Protein-Protein Interaction (PPI) network. (**C**) GO enrichment analysis and (**D**) KEGG enrichment analysis of the 128 potential targets. T Bubble size represents gene expression levels within individual pathways, while color saturation denotes the statistical significance of pathway enrichment. This pathway map was adapted from KEGG and is reproduced with permission from Kanehisa Laboratories under KEGG Copyright Permission Reference No. 254,190^33^.

Potential biological targets of PET were systematically predicted using the SwissTargetPrediction and Similarity Ensemble Approach (SEA) databases. Following data integration, a total of 128 unique potential targets were identified (Fig. 2A). The identified targets were imported into the STRING database to generate a protein-protein interaction (PPI) network, with subsequent visualization performed using Cytoscape software (Fig. 2B).

The 128 candidate targets were systematically analyzed through DAVID-based functional enrichment, identifying 144 KEGG pathways and 509 GO annotations for *Homo sapiens*. Functional stratification yielded 320 biological process terms, 52 cellular component terms, and 137 molecular function terms, with enrichment significance assessed by *p* values. Top-ranked GO terms (Fig. 2C) were prioritized based on enrichment significance and gene ratio, and visualized using bubble plots. KEGG pathway prioritization (Fig. 2D) employed identical significance thresholds.

Analysis of BP terms (Fig. 2C) revealed enrichment in signal transduction, proteolysis, apoptotic process, chromatin remodeling, and other related processes. The enriched CC terms were predominantly associated with the cytosol, plasma membrane, cytoplasm, nucleus, and other related structures. The MF category was predominantly enriched in terms related to protein binding, ATP binding, identical protein binding, metal ion binding, and other related molecular functions. Analysis of KEGG terms (Fig. 2D) revealed enrichment in MAPK signaling pathway, TNF signaling pathway, and other related processes.

### Identification of CD-associated targets

**Fig. 3:**
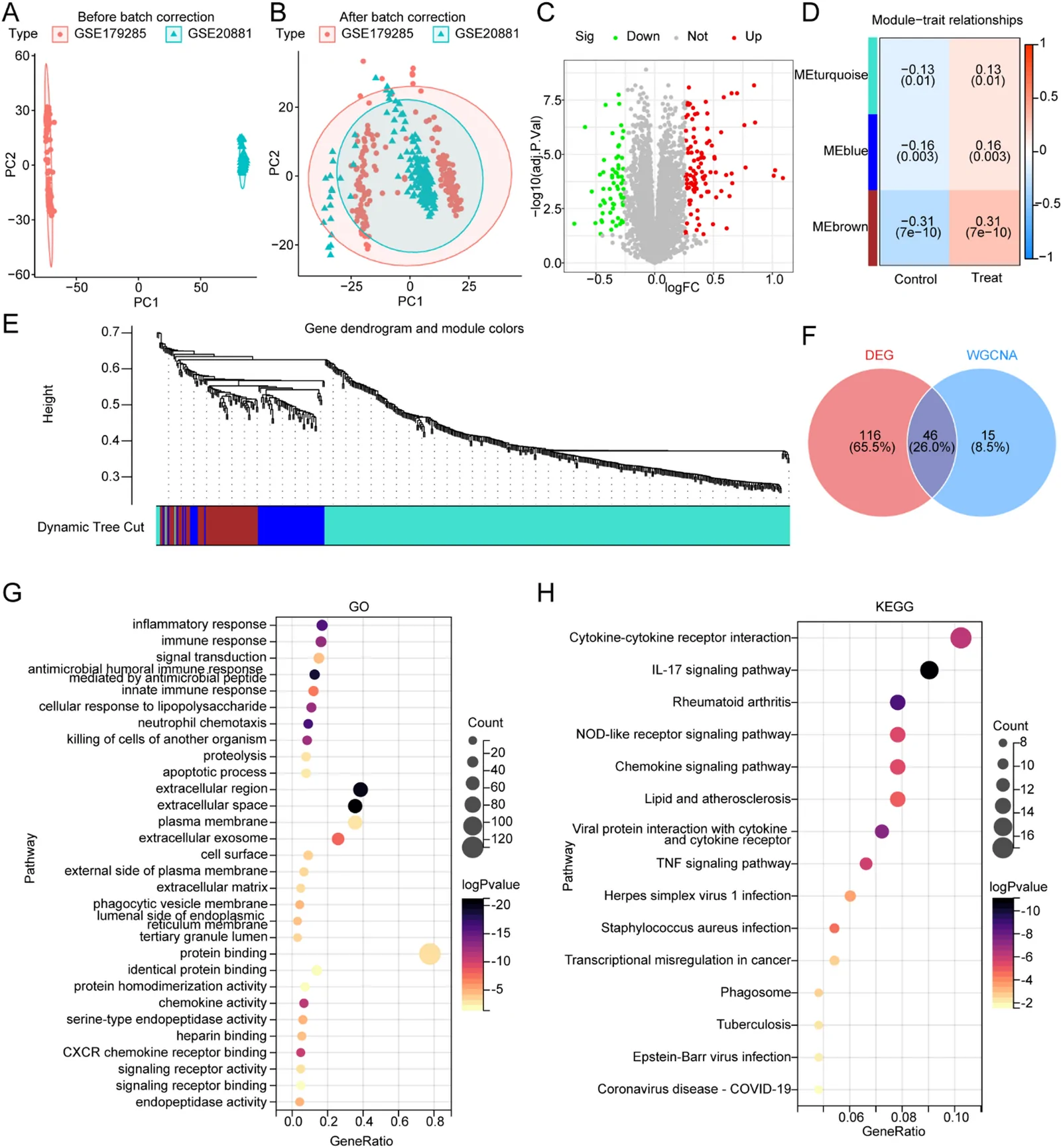
Identification of the targets for CD. (**A**) PCA scatter plot of the GSE179285 and GSE20881 datasets before batch correction. (**B**) PCA scatter plot of the GSE179285 and GSE20881 datasets after batch correction. (**C**) Volcano plot displaying differentially expressed genes (DEGs). (**D**) Module-trait relationships heatmap. (**E**) Gene dendrogram generated by WGCNA. (**F**) Venn diagram of differentially expressed genes (DEGs) and WGCNA module genes. (**G**) GO enrichment analysis and (**H**) KEGG enrichment analysis of the 177 targets. Bubble size represents gene expression levels within individual pathways, while color saturation denotes the statistical significance of pathway enrichment. This pathway map was adapted from KEGG and is reproduced with permission from Kanehisa Laboratories under KEGG Copyright Permission Reference No. 254,190^33^.

To mitigate batch effects, the GSE179285 and GSE20881 datasets were merged, followed by comprehensive normalization of the gene expression matrix. PCA showed a clear reduction in dispersion of the data distribution after normalization compared to pre-normalized data (Fig. 3A and B). Differential expression analysis identified 162 significantly altered genes, visualized through a volcano plot (Fig. 3C).

WGCNA identified three distinct gene modules (Fig. 3E). Module-trait relationship analysis revealed statistically significant associations (*P* < 0.05) between all identified modules and CD (Fig. 3D). Integration of DEGs with genes from the brown WGCNA module yielded a consolidated set of 177 CD-associated genes (Fig. 3F).

The 117 candidate targets were systematically analyzed through DAVID-based functional enrichment, identifying 46 KEGG pathways and 181 GO annotations for *Homo sapiens*. Functional stratification yielded 111 biological process terms, 26 cellular component terms, and 44 molecular function terms, with enrichment significance assessed by *p* values. Top-ranked GO terms (Fig. 3G) were prioritized based on enrichment significance and gene ratio, and visualized using bubble plots. KEGG pathway prioritization (Fig. 3H) employed identical significance thresholds.

Analysis of BP terms (Fig. 3G) revealed enrichment in inflammatory response, immune response, signal transduction, and other related processes. The enriched CC terms were predominantly associated with the extracellular region, extracellular space, plasma membrane, extracellular exosome, and other related structures. The MF category was predominantly enriched in terms related to protein binding, identical protein binding, protein homodimerization activity, chemokine activity, and other related molecular functions. Analysis of KEGG terms (Fig. 3H) revealed enrichment in IL-17 signaling pathway, NOD-like receptor signaling pathway, TNF signaling pathway, and other related processes.

### Identification of PET-associated disease targets in CD

**Fig. 4:**
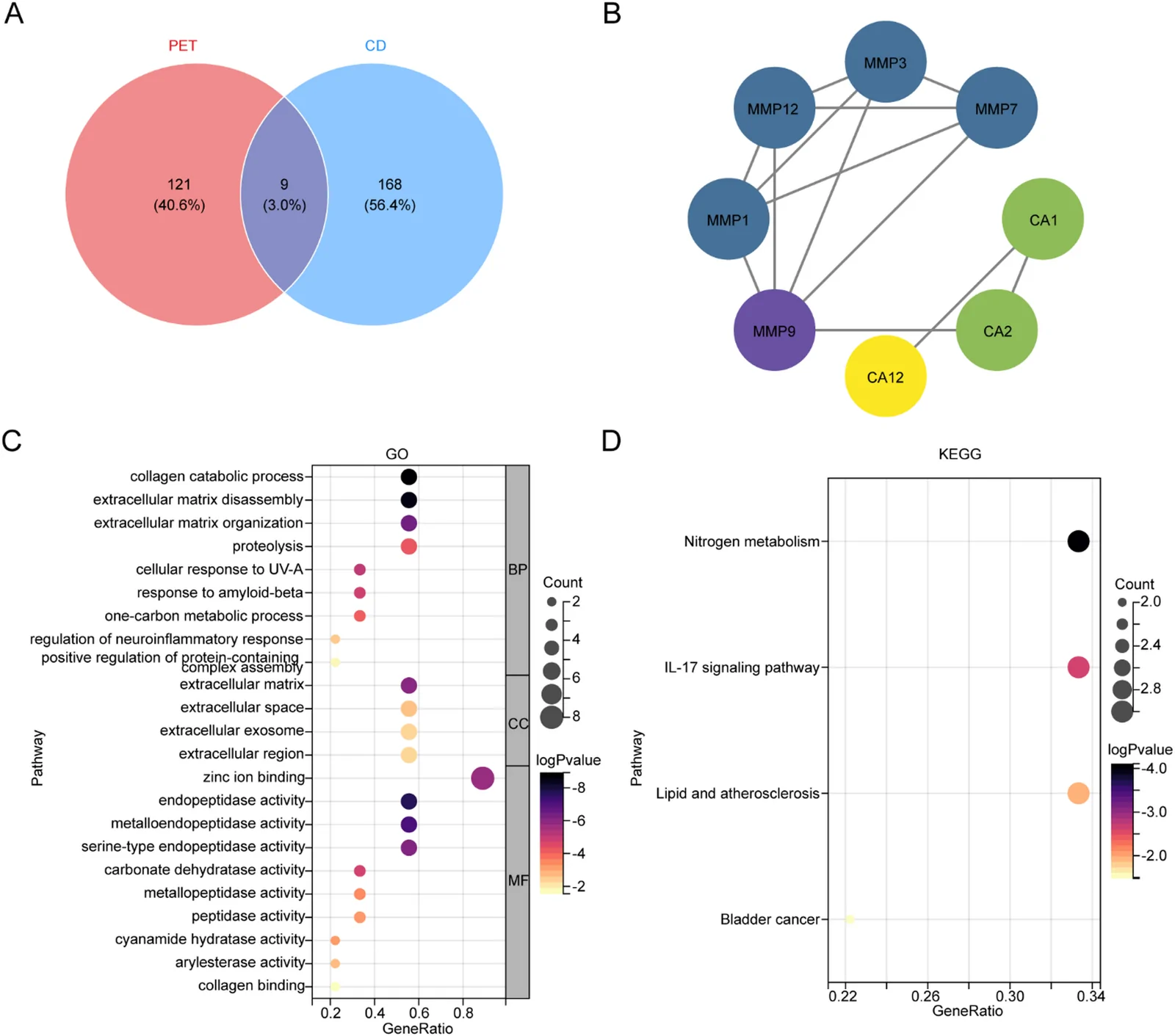
Identification of PET-associated disease targets in CD. (**A**) Venn diagram of PET-associated genes and CD-related genes. (**B**) Visualization of overlapping genes interactions using a Protein-Protein Interaction (PPI) network. (**C**) GO enrichment analysis and (**D**) KEGG enrichment analysis of the 9 targets. Bubble size represents gene expression levels within individual pathways, while color saturation denotes the statistical significance of pathway enrichment. This pathway map was adapted from KEGG and is reproduced with permission from Kanehisa Laboratories under KEGG Copyright Permission Reference No. 254,190^33^.

Intersection analysis between PET targets and CD-associated genes identified 9 potential targets (Fig. 4A). The identified targets were imported into the STRING database to generate a protein-protein interaction (PPI) network, with subsequent visualization performed using Cytoscape software (Fig. 4B).

The 9 candidate targets were systematically analyzed through DAVID-based functional enrichment, identifying 8 KEGG pathways and 24 GO annotations for *Homo sapiens*. Functional stratification yielded 10 biological process terms, 4 cellular component terms, and 10 molecular function terms, with enrichment significance assessed by *p* values. Top-ranked GO terms (Fig. 4C) were prioritized based on enrichment significance and gene ratio, and visualized using bubble plots. KEGG pathway prioritization (Fig. 4D) employed identical significance thresholds.

Analysis of BP terms (Fig. 4C) revealed enrichment in collagen catabolic process, extracellular matrix disassembly, extracellular matrix organization, and other related processes. The enriched CC terms were predominantly associated with the extracellular matrix, extracellular space, extracellular exosome, and extracellular region. The MF category was predominantly enriched in terms related to zinc ion binding, endopeptidase activity, metalloendopeptidase activity, serine-type endopeptidase activity, and other related molecular functions. Analysis of KEGG terms (Fig. 4D) revealed enrichment in the IL-17 signaling pathway, the Lipid and atherosclerosis pathway, and other related pathways.

### Identification of PET-associated hub targets in CD

**Fig. 5:**
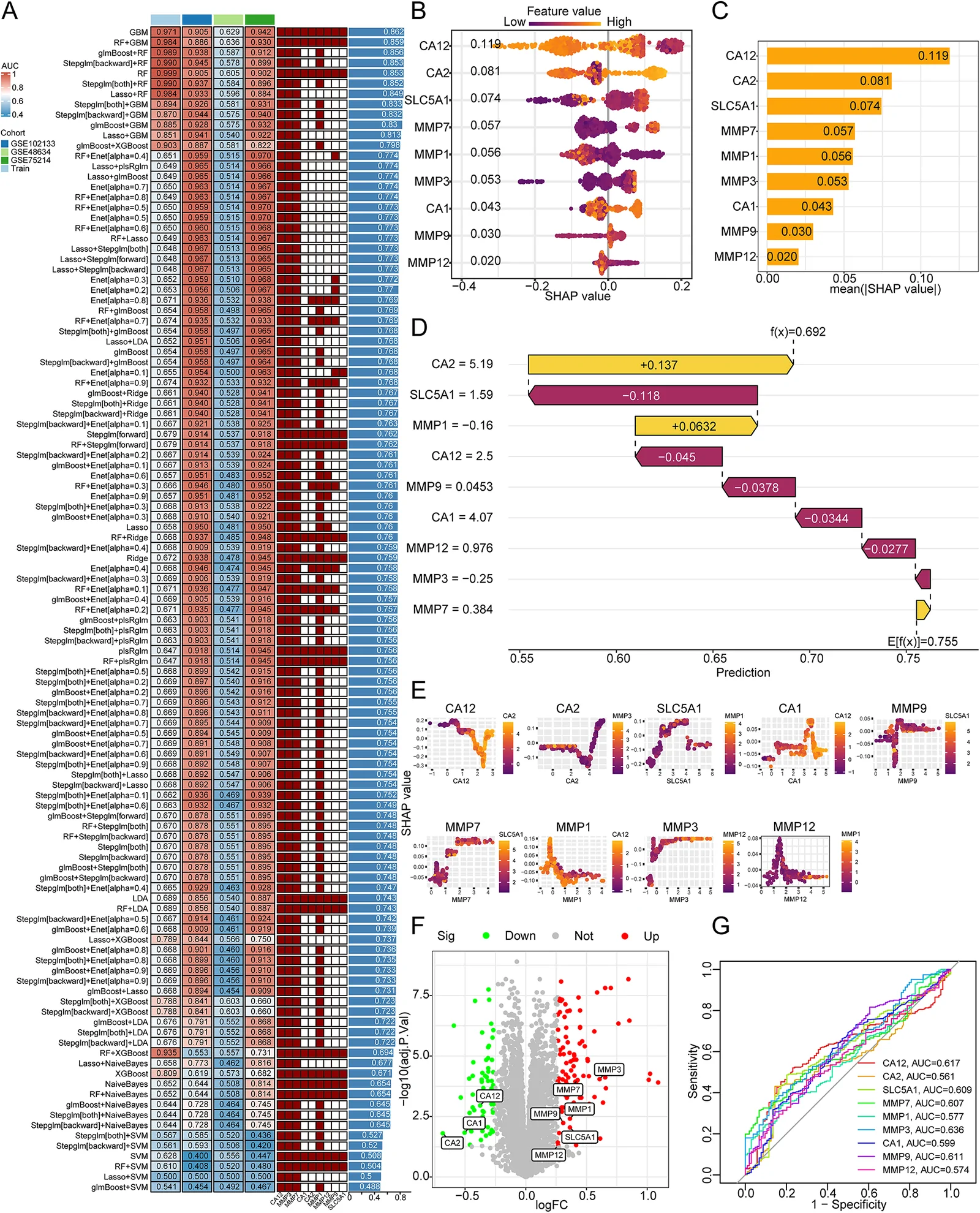
Identification of hub genes in PET-induced CD. (**A**) Heatmap displaying AUC values of different models across multiple cohorts. Left column: Model names. Middle column: Selected gene features for each model. Right column: Mean AUC values. (**B**) SHAP analysis visualizations including beeswarm plot, (**C**) bar plot, (**D**) waterfall plot, and (**E**) dependence plot. (**C**) Annotation map of cell types. (**F**) Volcano plot marking critical regulatory genes. (**G**) ROC curves evaluating the diagnostic performance of key genes.

Using multiple machine learning algorithms, we constructed 113 models to identify hub genes among the 9 candidate targets associated with PET-MPs-related CD. The Gradient Boosting Machine (GBM) model achieved the best predictive performance, with the highest mean ROC-AUC across both training and validation cohorts (Fig. 5A), and the following nine genes were identified as the most important features: CA12, CA2, SLC5A1, MMP7, MMP1, MMP3, CA1, MMP9, and MMP12. SHAP analysis assessed the contribution of each gene to the model output, with CA12 (mean |SHAP| = 0.119), CA2 (0.081), and SLC5A1 (0.074) exhibiting the highest influence (Fig. 5B and C). Waterfall plot analysis showed that SLC5A1 (feature value = 1.59, SHAP Δ = − 0.118) was associated with a negative contribution to the prediction, whereas CA2 (feature value = 5.19, Δ = 0.137) was associated with a positive contribution. The combined effect resulted in a predicted probability (f(x) = 0.692) below the baseline expectation (E[f(x)] = 0.755) (Fig. 5D). Dependence plot analysis indicated that CA12 expression was positively associated with CA2 and CA1 expression levels. Furthermore, the predictive contribution of CA2 peaked at high expression levels, whereas that of CA1, MMP1, and MMP12 was optimal at moderate expression levels (Fig. 5E). The differential expression patterns of these genes in CD tissues were visualized using a volcano plot (Fig. 5F). Notably, ROC curve analysis showed that these hub genes exhibited limited individual diagnostic performance (AUC < 0.7, Fig. 5G); however, their prioritization was primarily driven by their high importance in the machine learning model and network topological features, rather than stand-alone diagnostic accuracy.

### Identification of UC-associated targets

**Fig. 6:**
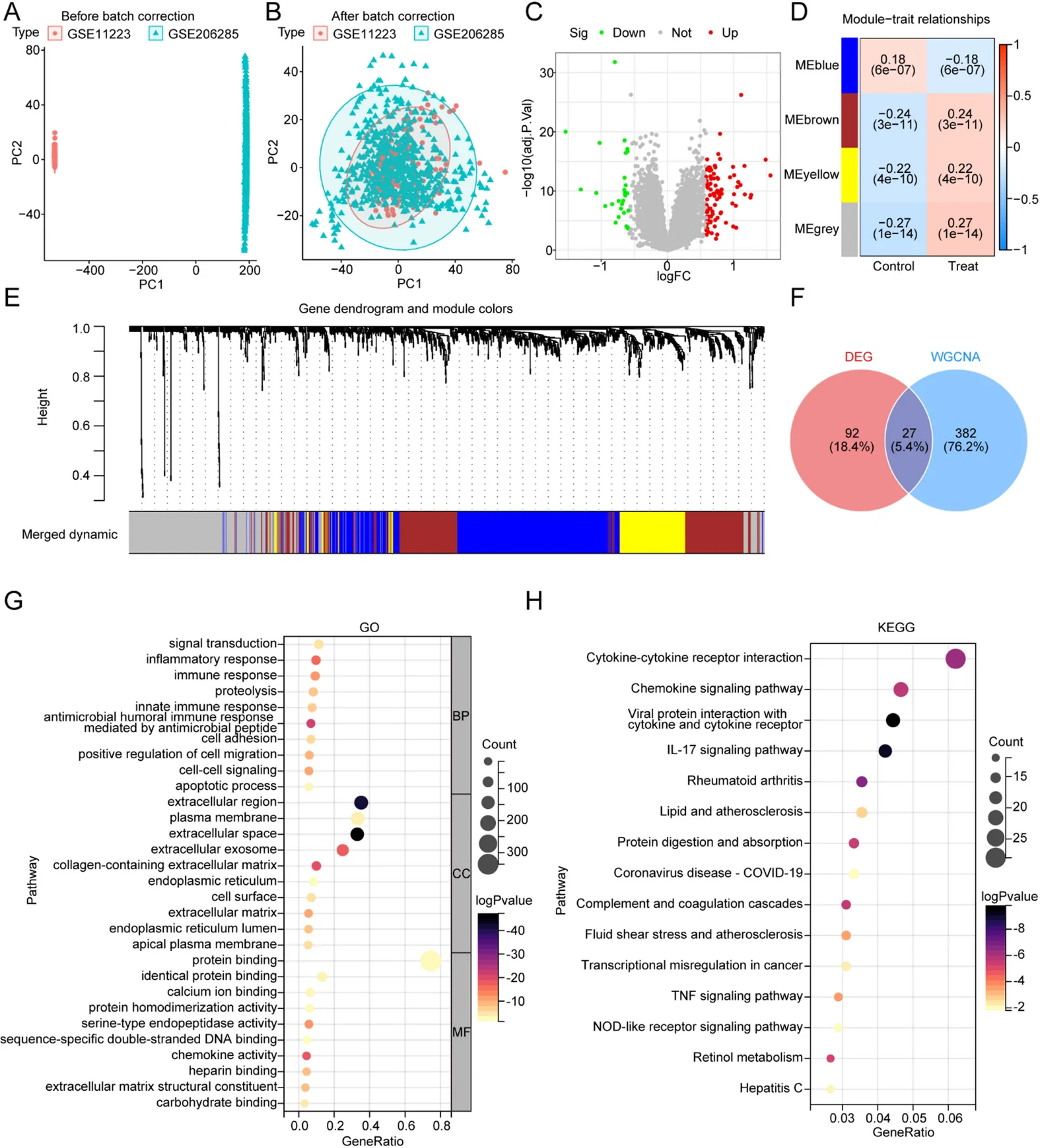
Identification of the targets for UC. (**A**) PCA scatter plot of the GSE11223 and GSE206285 datasets before batch correction. (**B**) PCA scatter plot of the GSE11223 and GSE206285 datasets after batch correction. (**C**) Volcano plot displaying differentially expressed genes (DEGs). (**D**) Module-trait relationships heatmap. (**E**) Gene dendrogram generated by WGCNA. (**F**) Venn diagram of differentially expressed genes (DEGs) and WGCNA module genes. (**G**) GO enrichment analysis and (**H**) KEGG enrichment analysis of the 501 targets. Bubble size represents gene expression levels within individual pathways, while color saturation denotes the statistical significance of pathway enrichment. This pathway map was adapted from KEGG and is reproduced with permission from Kanehisa Laboratories under KEGG Copyright Permission Reference No. 254,190^33^.

To mitigate batch effects, the GSE11223 and GSE206285 datasets were merged, followed by comprehensive normalization of the gene expression matrix. PCA showed a clear reduction in dispersion of the data distribution after normalization compared to pre-normalized data (Fig. 6A and B). Differential expression analysis detected 119 significantly altered genes, visualized in a volcano plot (Fig. 6C).

WGCNA identified four distinct gene modules (Fig. 6E). Module-trait relationship analysis revealed statistically significant associations (*P* < 0.05) between all identified modules and UC (Fig. 6D). Integration of DEGs with genes from the grey WGCNA module yielded a consolidated set of 501 UC-associated genes (Fig. 6F).

The 501 candidate targets were systematically analyzed through DAVID-based functional enrichment, identifying 49 KEGG pathways and 385 GO annotations for *Homo sapiens*. Functional stratification yielded 265 biological process terms, 39 cellular component terms, and 81 molecular function terms, with enrichment significance assessed by *p* values. Top-ranked GO terms (Fig. 6G) were prioritized based on enrichment significance and gene ratio, and visualized using bubble plots. KEGG pathway prioritization (Fig. 6H) employed identical significance thresholds.

Analysis of BP terms (Fig. 6G) revealed enrichment in signal transduction, inflammatory response, immune response, and other related processes. The enriched CC terms were predominantly associated with the extracellular region, plasma membrane, extracellular space, extracellular exosome, and other related structures. The MF category was predominantly enriched in terms related to protein binding, identical protein binding, calcium ion binding, protein homodimerization activity, and other related molecular functions. Analysis of KEGG terms (Fig. 6H) revealed enrichment in IL-17 signaling pathway, TNF signaling pathway, NOD-like receptor signaling pathway, and other related pathways.

### Identification of PET-associated disease targets in UC

**Fig. 7:**
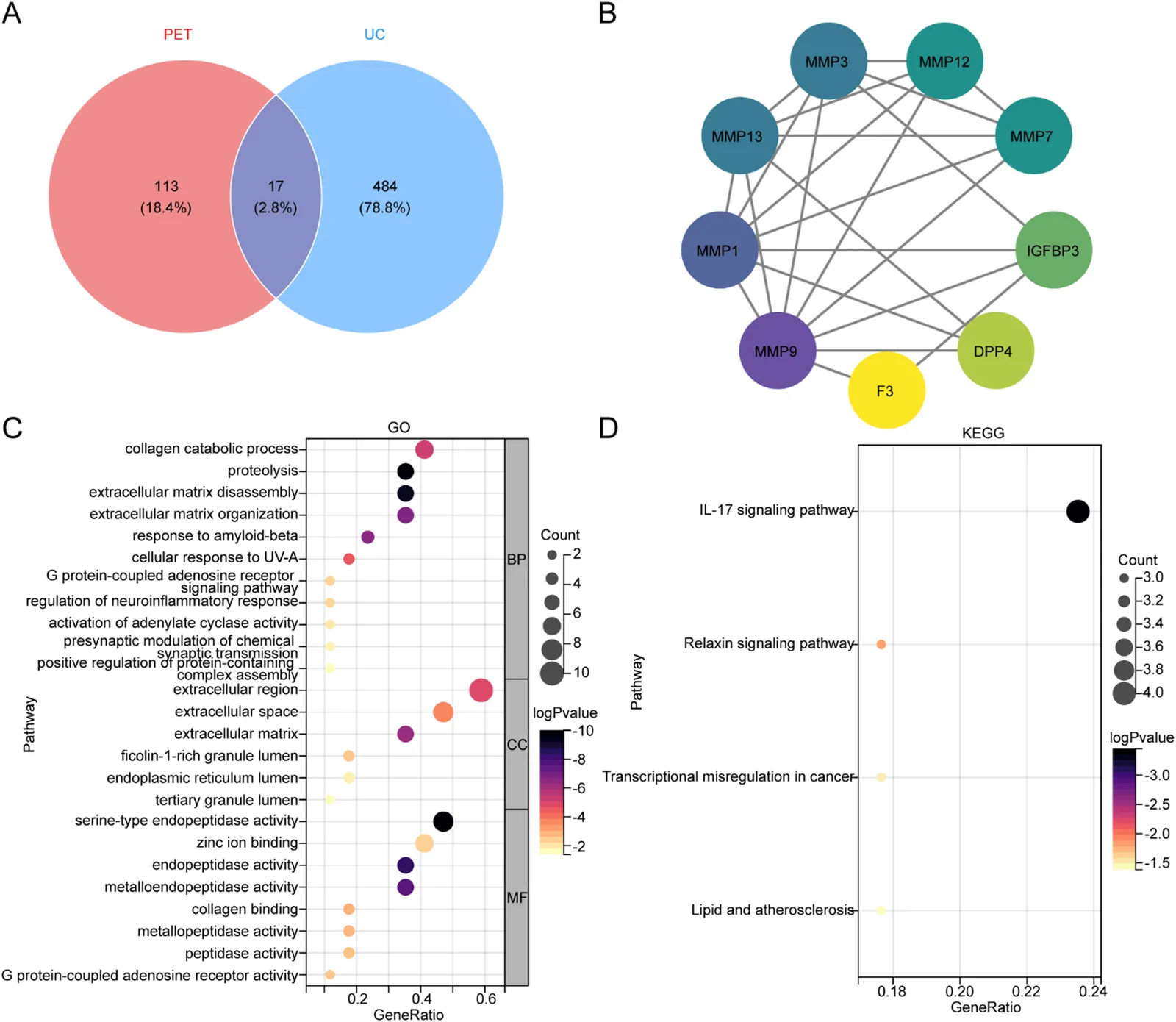
Identification of PET-associated disease targets in UC. (**A**) Venn diagram of PET-associated genes and UC-related genes. (**B**) Visualization of overlapping genes interactions using a Protein-Protein Interaction (PPI) network. (**C**) GO enrichment analysis and (**D**) KEGG enrichment analysis of the 17 targets. Bubble size represents gene expression levels within individual pathways, while color saturation denotes the statistical significance of pathway enrichment. This pathway map was adapted from KEGG and is reproduced with permission from Kanehisa Laboratories under KEGG Copyright Permission Reference No. 254,190^33^.

Intersection analysis between PET targets and UC-associated genes identified 17 potential targets (Fig. 7A). The identified targets were imported into the STRING database to generate a protein-protein interaction (PPI) network, with subsequent visualization performed using Cytoscape software (Fig. 7B).

The 17 candidate targets were systematically analyzed through DAVID-based functional enrichment, identifying 5 KEGG pathways and 28 GO annotations for *Homo sapiens*. Functional stratification yielded 11 biological process terms, 8 cellular component terms, and 9 molecular function terms, with enrichment significance assessed by *p* values. Top-ranked GO terms (Fig. 7C) were prioritized based on enrichment significance and gene ratio, and visualized using bubble plots. KEGG pathway prioritization (Fig. 7D) employed identical significance thresholds.

Analysis of BP terms (Fig. 7C) revealed enrichment in proteolysis, collagen catabolic process, extracellular matrix disassembly, and other related processes. The enriched CC terms were predominantly associated with the extracellular region, extracellular space, extracellular matrix, and other related structures. The MF category was predominantly enriched in terms related to serine-type endopeptidase activity, zinc ion binding, endopeptidase activity, metalloendopeptidase activity, and other related molecular functions. Analysis of KEGG terms (Fig. 7D) revealed enrichment in the IL-17 signaling pathway, the Lipid and atherosclerosis pathway, and other related pathways.

### Identification of PET-associated hub targets in UC

**Fig. 8:**
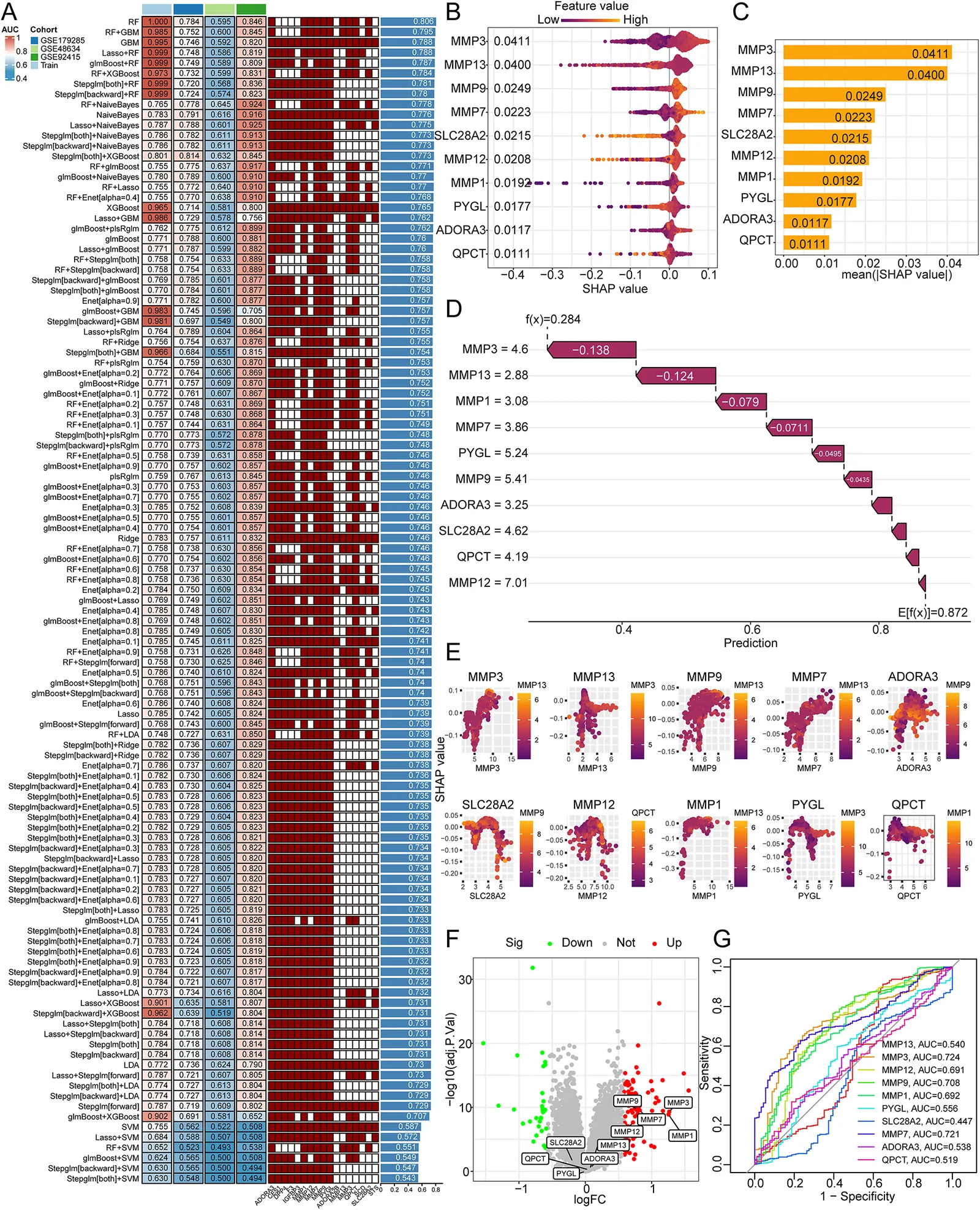
Identification of hub genes in PET-induced UC. (**A**) Heatmap displaying AUC values of different models across multiple cohorts. Left column: Model names. Middle column: Selected gene features for each model. Right column: Mean AUC values. (**B**) SHAP analysis visualizations including beeswarm plot, (**C**) bar plot, (**D**) waterfall plot, and (**E**) dependence plot. (**C**) Annotation map of cell types. (**F**) Volcano plot marking critical regulatory genes. (**G**) ROC curves evaluating the diagnostic performance of key genes.

Using multiple machine learning algorithms, we constructed 113 models to identify hub genes among the 17 candidate targets associated with PET-MPs-related UC. The Random Forest (RF) model achieved the best predictive performance, with the highest mean ROC-AUC across both training and validation cohorts (Fig. 8A), and the following ten genes were identified as the most important features: MMP3, MMP13, MMP9, MMP7, SLC28A2, MMP12, MMP1, PYGL, ADORA3, and QPCT. SHAP analysis assessed the contribution of each gene to the model output, with MMP3 (mean |SHAP| = 0.0411) and MMP13 (0.0400) exhibiting the highest influence (Fig. 8B and C). Waterfall plot analysis indicated that MMP3 (feature value = 4.6, SHAP Δ = − 0.138) and MMP13 (feature value = 2.88, Δ = − 0.124) were associated with a negative contribution to the prediction (Fig. 8D). Their combined negative contributions resulted in a predicted probability (f(x) = 0.284) below the baseline expectation (E[f(x)] = 0.872). Dependence plot analysis indicated that the predictive contribution of MMP3, MMP12, SLC28A2, and ADORA3 was optimal at moderate expression levels (Fig. 8E). The differential expression patterns of these 10 key genes in UC tissues were visualized using a volcano plot (Fig. 8F). ROC curve analysis showed that MMP3, MMP9, and MMP7 exhibited strong individual diagnostic performance (AUC > 0.70), whereas the remaining hub genes demonstrated limited discriminatory ability (AUC < 0.70, Fig. 8G); their prioritization was primarily driven by their high feature importance in the RF model rather than stand-alone diagnostic accuracy.

### Molecular docking validation of PET-hub target interactions

**Fig. 9:**
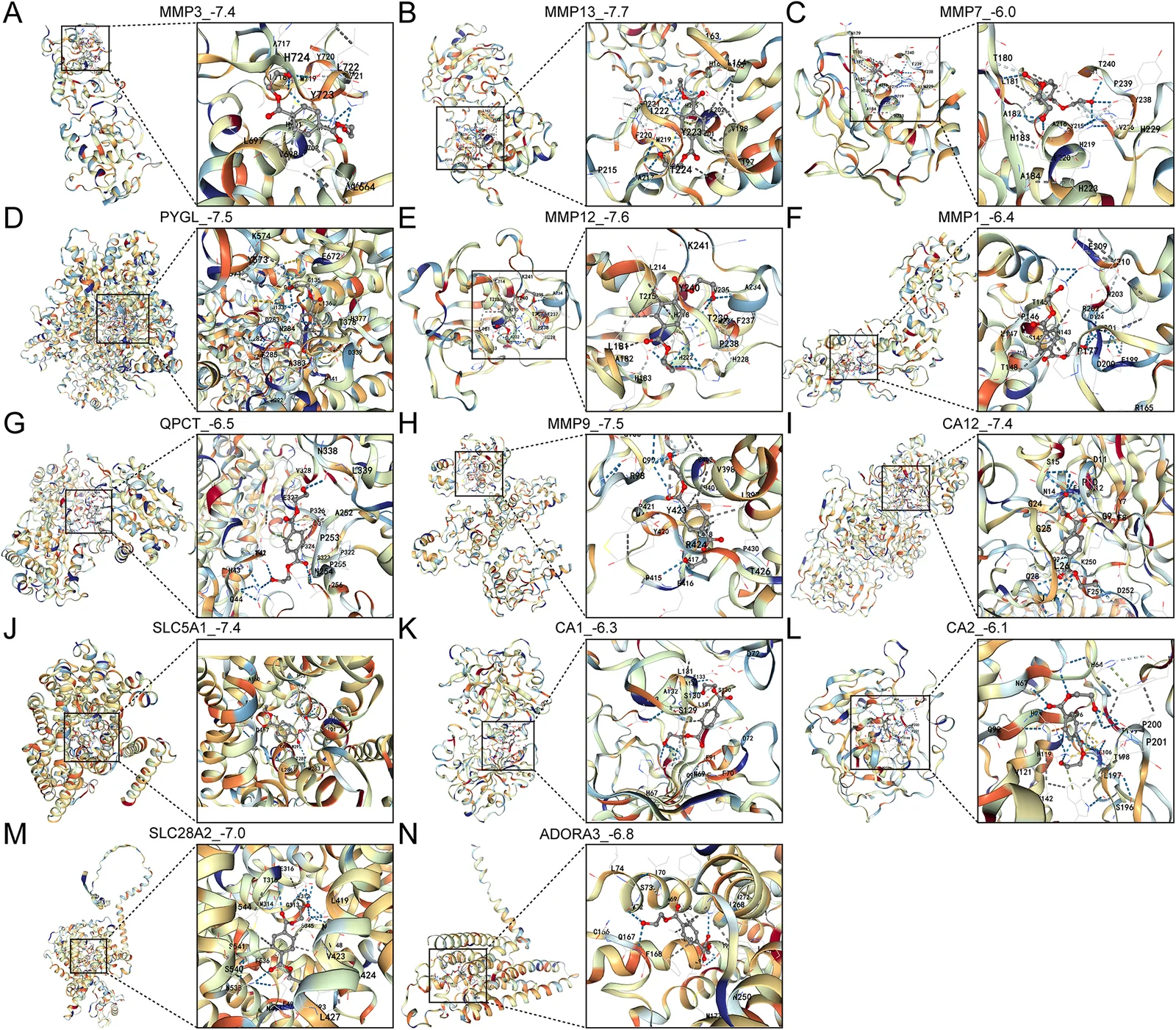
Molecular docking between hub targets of IBD and PET. (**A**) The docking results indicate that PET interacts with MMP3, (**B**) MMP13, (**C**) MMP7, (**D**) PYGL, (**E**) MMP12, (**F**) MMP1, (**G**) QPCT, (**H**) MMP9, (**I**) CA12, (**J**) SLC5A1, (**K**) CA1, (**L**) CA2, (**M**) SLC28A2, and (**N**) ADORA3.

Molecular docking simulations using a PET-related compound as a proxy predicted binding interactions with the 14 hub targets (MMP3, MMP13, MMP7, PYGL, MMP12, MMP1, QPCT, MMP9, CA12, SLC5A1, CA1, CA2, SLC28A2, and ADORA3) associated with IBD. The results showed favorable binding affinities (all ≤ − 6 kcal/mol), suggesting potentially stable and spontaneous interactions. The predicted binding poses for each compound–protein complex are shown in Fig. 9. Collectively, these computational findings suggest that the PET-related compound can engage in high-affinity interactions with the IBD-relevant hub targets prioritized by our machine learning framework, providing a structural basis for further investigation.

## Discussion

This study employed a network toxicology approach, integrating data from the GEO, SEA, SwissTargetPrediction, and PDB databases. Combined with multiple machine learning methods, we identified 14 hub genes that were computationally prioritized as candidate mediators linking PET-MPs exposure to IBD.

MMP3, MMP13, MMP7, MMP12, MMP1, and MMP9 belong to the matrix metalloproteinase (MMP) family. Current evidence suggests that MMPs may contribute to IBD pathogenesis by promoting immune cell infiltration and modulating immune responses^32^. As members of the carbonic anhydrase (CA) family, CA1, CA2, and CA12 are known to regulate fundamental physiological processes, including acid-base homeostasis and electrolyte balance. While these enzymes represent mechanistically plausible candidates for IBD pathogenesis, empirical validation of their disease relevance remains outstanding. In IBD, QPCT (glutaminyl-peptide cyclotransferase) may participate in inflammatory protein modification or drug metabolism, while SLC28A2 (solute carrier family 28 member 2) could influence intestinal epithelial regeneration through nucleotide supply. SLC5A1 (solute carrier family 5 member 1) may contribute to nutritional metabolic dysregulation, though conclusive evidence for these mechanisms is currently lacking. Although ADORA3 (adenosine A3 receptor) agonists alleviate colitis in animal models, human genetic association studies in IBD have not yet reported significant links. The role of PYGL (glycogen phosphorylase L) in IBD remains undefined.

We employed 12 classical machine learning algorithms to construct 113 machine learning prediction models. For CD, 11 models demonstrated a mean ROC-AUC greater than 0.8 across both the training and validation sets. For UC, although only one model achieved a mean ROC-AUC above 0.8, 39 models still attained a mean ROC-AUC greater than 0.75. These results suggest that the constructed models may offer useful predictive performance for identifying candidate gene targets. Subsequently, SHAP analysis was used to evaluate the relative contributions of each gene to the model predictions.

The accumulation of diverse microplastic particles in the environment complicates their adverse effects on human health. Mitigating microplastic pollution is therefore important for reducing potential health risks. Prior to addressing these challenges, the safe recycling and disposal of plastics should be prioritized to ameliorate environmental contamination. Concurrently, public education on the potential risks of microplastics and the promotion of eco-conscious consumption can also play important roles in reducing human exposure.

This research suggests that integrating network toxicology with machine learning may provide a useful strategy for exploring the potential molecular mechanisms of PET-MPs. By combining machine learning, genomics, and network toxicology, we established a computational framework for the rapid and systematic prioritization of candidate molecular targets potentially associated with PET-MPs exposure. Molecular docking provided complementary insights into the potential binding interactions between a PET-related compound and the hub targets, offering structural plausibility for the predicted associations. Our results, derived from computational simulations, cannot fully capture the complexity of long-term, real-world human PET-MPs exposure scenarios. Future studies should prioritize experimental validation of identified molecular targets and biological pathways using integrative in vitro (cell models) and in vivo (animal models) approaches. Collectively, this work provides a hypothesis-generating framework for further investigation into the potential role of PET-MPs in IBD pathogenesis, which may ultimately inform the development of preventive and therapeutic strategies.

## Limitations and future directions

Several limitations of this study should be acknowledged. First, our findings are derived entirely from computational predictions and publicly available datasets; no experimental validation was performed. Therefore, all identified hub genes, molecular pathways, and docking interactions represent hypothesis‑generating results and must be interpreted with caution. In particular, the molecular docking simulations employed a small‑molecule proxy for PET‑MPs, which cannot recapitulate the interfacial complexity or particulate nature of real microplastics, and the binding predictions require experimental confirmation. Second, the datasets used in this study are subject to inherent batch effects, limited sample sizes, and population heterogeneity, which may influence the generalizability of the predictive models. Third, the modest individual diagnostic performance of several hub genes (AUC < 0.7) suggests that they are primarily prioritized by model contribution rather than stand‑alone discriminatory power, and their biological relevance remains to be established.

To build upon these computational findings, future studies should incorporate rigorous experimental strategies. In vitro validation using relevant intestinal epithelial cell lines (e.g., Caco‑2 or HT‑29) exposed to PET‑MP particles is necessary to assess the expression changes of the identified hub genes and proteins by qPCR and western blotting. Additionally, gene knockdown or overexpression experiments could help define their functional roles in PET‑MP‑triggered inflammatory responses. In vivo animal models of colitis exposed to environmentally relevant concentrations of PET‑MPs would be instrumental in evaluating the systemic and local effects on intestinal inflammation, as well as in verifying the predicted target interactions. Ultimately, integrating these experimental approaches with the computational framework established here will be essential to translate our predictive observations into a mechanistic understanding of PET‑MP‑associated IBD pathogenesis.

## Conclusion

This study suggests that PET-MPs may contribute to the pathogenesis of Crohn’s disease (CD) and ulcerative colitis (UC) by potentially interacting with 14 computationally prioritized hub genes—MMP3, MMP13, MMP7, PYGL, MMP12, MMP1, QPCT, MMP9, CA12, SLC5A1, CA1, CA2, SLC28A2, and ADORA3—and through the modulation of two pathways: the IL-17 signaling pathway and the Lipid and atherosclerosis pathway. Molecular docking simulations using a PET-related compound indicated favorable binding affinities with these hub targets, suggesting potential interactions. These findings provide a hypothesis-generating framework for further investigation into the potential roles of PET-MPs in CD and UC pathogenesis. Future studies should aim to validate the predicted associations and examine whether PET-MPs exposure plays a causal role in IBD, while also exploring interventions that may alleviate their potential impact on intestinal homeostasis.

## Supplementary Information

Below is the link to the electronic supplementary material.


Supplementary Material 1


## Data Availability

The datasets used and analysed during the current study are available in the [GEO] repository, (https://www.ncbi.nlm.nih.gov/geo/)].
