# Supplementary material for: Investigation of the molecular network underlying PET-MPs-induced inflammatory bowel disease via integrated machine learning and molecular docking approaches
Source: Sci Rep. 2026 Jun 14;16:27152. doi: 10.1038/s41598-026-57094-0 (PMC13526889; doi:10.1038/s41598-026-57094-0)
Supplement: Supplementary file 1 [file 41598_2026_57094_MOESM1_ESM.docx]

## Table S1

|  | GEO accession number | platform | sample size | | usage |
| --- | --- | --- | --- | --- | --- |
|  |  |  | normal | disease |  |
| Crohn's disease | GSE20881 | GPL1708 | 73 | 99 | discovery cohort |
|  | GSE179285 | GPL6480 | 31 | 168 |  |
|  | GSE48634 | GPL10558 | 69 | 34 | validation cohort |
|  | GSE75214 | GPL6244 | 22 | 75 | validation cohort |
|  | GSE102133 | GPL6244 | 12 | 65 | validation cohort |
| Ulcerative colitis | GSE11223 | GPL1708 | 73 | 129 | discovery cohort |
|  | GSE206285 | GPL13158 | 18 | 550 |  |
|  | GSE48634 | GPL10558 | 69 | 68 | validation cohort |
|  | GSE92415 | GPL13158 | 21 | 162 | validation cohort |
|  | GSE179285 | GPL6480 | 31 | 55 | validation cohort |
